# Environmental and genetic determinants of plasmid mobility in pathogenic *Escherichia coli*

**DOI:** 10.1126/sciadv.aax3173

**Published:** 2020-01-24

**Authors:** Jonathan H. Bethke, Adam Davidovich, Li Cheng, Allison J. Lopatkin, Wenchen Song, Joshua T. Thaden, Vance G. Fowler, Minfeng Xiao, Lingchong You

**Affiliations:** 1 Department of Molecular Genetics and Microbiology, Duke University, Durham, NC 27708, USA.; 2 Department of Biomedical Engineering, Duke University, Durham, NC 27708, USA.; 3 BGI-Shenzhen, Shenzhen 518083, China.; 4 China National Genebank, BGI-Shenzhen, Shenzhen 518120, China.; 5 School of Biology and Biological Engineering, South China University of Technology, Guangzhou 510006, China.; 6 Department of Medicine, Division of Infectious Diseases, Duke University Medical Center, Durham, NC 27710, USA.; 7 Center for Genomic and Computational Biology, Duke University, Durham, NC 27708, USA.

## Abstract

Plasmids are key vehicles of horizontal gene transfer (HGT), mobilizing antibiotic resistance, virulence, and other traits among bacterial populations. The environmental and genetic forces that drive plasmid transfer are poorly understood, however, due to the lack of definitive quantification coupled with genomic analysis. Here, we integrate conjugative phenotype with plasmid genotype to provide quantitative analysis of HGT in clinical *Escherichia coli* pathogens. We find a substantial proportion of these pathogens (>25%) able to readily spread resistance to the most common classes of antibiotics. Antibiotics of varied modes of action had less than a 5-fold effect on conjugation efficiency in general, with one exception displaying 31-fold promotion upon exposure to macrolides and chloramphenicol. In contrast, genome sequencing reveals plasmid incompatibility group strongly correlates with transfer efficiency. Our findings offer new insights into the determinants of plasmid mobility and have implications for the development of treatments that target HGT.

## Teaser

Integrating genomics with growth-based phenotyping reveals divergent roles of antibiotics and incompatibility in plasmid mobility.

## INTRODUCTION

The spread of antibiotic resistance is outpacing the development of new antibiotics. On average, new antibiotics cost upward of $500 million USD and take 10 years to develop, only to have widespread resistance appear in less than 3 years (*1*, *2*). Horizontal gene transfer (HGT) is often implicated in this rapid decline in efficacy, mobilizing reservoirs of resistance long established in the environment by natural antibiotic producers (*3*). In this way, targeting mobilization to restore antibiotic efficacy may be more successful than trying to eliminate resistance outright (*4*, *5*).

Conjugation, the direct transfer of DNA from a donor to a recipient, is considered the HGT mechanism most responsible for mobilizing resistance among Enterobacteriaceae (*2*, *6*). Conjugative plasmids are exchanged across broad host ranges, harbor resistance to virtually all antibiotics, and may be maintained in hosts with little cost, or in spite of costs, to fitness (*7*, *8*). Furthermore, resistance genes tend to cluster on plasmids, allowing acquisition of multidrug resistance in a single transfer and making antibiotic selection for one now select for all (*2*).

**Fig. 1:**
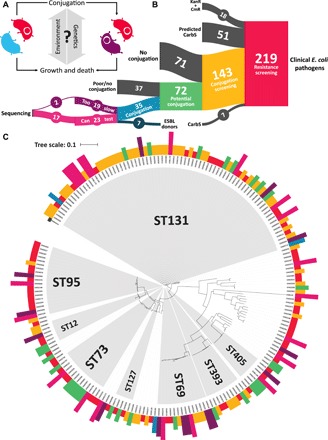
The environmental and genetic determinants of plasmid mobility. (**A**) The influence of environmental and genetic factors on conjugation is confounded by growth dynamics between donor (red), recipient (blue), and transconjugant (purple) populations. By decoupling conjugation modulation from growth dynamics, we can identify and use the determinants of plasmid mobility to fight antibiotic resistance. (**B**) Assembling a library of natural isolates with quantifiable rates of conjugation is a substantial undertaking. Starting from a library of 219 clinical *E. coli* pathogens from patient bloodstream infections, we screened for the ability to transfer β-lactam resistance commonly found on plasmids native to the Enterobacteriaceae family. Approximately 25% of the carbenicillin-resistant (CarbR) isolates exhibited detectable transfer to chromosomally kanamycin (KanR)– or chloramphenicol (CmR)–resistant recipients. These and seven extended spectrum β-lactamase (ESBL) donors were subsequently used to examine environmental and genetic determinants of plasmid mobility. (**C**) The diversity present in the *E. coli* pathogen library is maintained through conjugation screening. A phylogenetic tree of the library was constructed from 200 genome assemblies (BioProject accession nos. PRJNA290784 and PRJNA551684) to reveal the breadth of our analysis throughout each phase of screening. Genome assemblies for the remaining 19 isolates were either unavailable or of insufficient quality. *E. coli* strain EC958 (GenBank accession no. HG941718.1) was used as a reference genome for alignment. Isolates are color labeled by their final phase. Major multilocus sequence types (≥5 isolates in common) present in the library are highlighted in gray.

Modulating conjugation to curtail the spread and maintenance of resistance requires an understanding of the environmental and genetic factors underlying plasmid mobility. Multiple factors have been proposed, including nutrient levels (*9*, *10*), cell-cell signaling (*11*–*13*), and even antibiotics (*11*, *14*–*17*), potentially accelerating the spread of resistance. Entire environments (e.g., animals, sewage, and biofilms) have also been labeled conjugative “hotspots,” uniting compatible donors, recipients, and plasmids under conditions that encourage HGT (*6*). However, growth dynamics are often coupled with conjugation modulation, making conjugation-specific effects difficult to interpret (Fig. 1A). For instance, in contrast to the prevailing conclusion that antibiotics promote conjugation, a recent study found no notable antibiotic effects when controlling for growth dynamics (*9*). Together with limited strain diversity and low-resolution genomic analysis, this ambiguity in conjugation quantification leaves the determinants of plasmid mobility in question.

To address these issues, we carried out the largest-scale analysis of conjugation phenotype paired with genotype in clinical *Escherichia coli* pathogens known for plasmid-borne multidrug resistance. In doing so, we introduce a new method for quantifying conjugation phenotype that increases throughput and sensitivity while reducing ambiguity due to growth dynamics. In general, we find that antibiotics exert little to no effect on conjugation efficiency, with one exception displaying significant promotion in the presence of macrolides and chloramphenicol (Cm). Conversely, conjugation efficiency strongly correlates with plasmid maintenance as indicated by the incompatibility (Inc) group. As our understanding of the environmental and genetic determinants of conjugation efficiency improves, modulation of HGT may become an important new tool for the fight against antibiotic resistance and control of bacterial evolution at large.

## RESULTS

### Conjugation in clinical *E. coli* pathogens

To study conjugation modulation, we focus on a library of 219 *E. coli* isolates collected from patient bloodstream infections at Duke University Hospital over 2002 to 2014. The Enterobacteriaceae family of Gram-negative bacteria serves as an ideal model to study HGT. Worldwide, it causes hundreds of millions of infections per year and harbors plasmids bearing multidrug resistance, most notably extended-spectrum β-lactamases (ESBLs) that cleave the most widely used class of antibiotics (*18*). Of these 219 isolates, 197 were chosen at random from the Duke Bloodstream Infection Biorepository (BSIB), and the remaining 22 were chosen for multidrug resistance.

We first screened the isolates for kanamycin (Kan) and Cm susceptibility to contrast with established MG1655 *E. coli* plasmid recipients with Kan or Cm resistance (Fig. 1B). All but 18 (8%) isolates were susceptible to at least one antibiotic, with 69 (32% total, 25% random) exhibiting resistance to Kan (50 μg/ml) and 31 (14% total, 13% random) exhibiting resistance to Cm (50 μg/ml). With Kan or Cm susceptibility established for each isolate, we screened for the ability to transfer β-lactam resistance to susceptible recipient *E. coli*. Of the Kan- or Cm-susceptible isolates, 143 were found to be resistant to carbenicillin (100 μg/ml; Carb). Clinical data from the BSIB indicating susceptibility to β-lactam antibiotics other than Carb were used to infer Carb susceptibility for 51 isolates. The 143 Carb-resistant isolates were then tested for the ability to generate Carb- and Cm/Kan-resistant transconjugants under dual-antibiotic selection. Subsequent rounds of dilution and repeat selection were performed to ensure that any growth under dual selection was from transconjugants, instead of donors or recipients. Of the 143 Carb-resistant isolates tested, 35 (24% total, 25% random) were capable of producing Carb- and Cm/Kan-resistant transconjugants under the experimental conditions. Phylogenetic analysis revealed that much of the genetic diversity present in the starting library was maintained throughout screening (Fig. 1C). Only plasmid-free recipients were used to prevent incompatibility issues with unknown isolate plasmids and retrotransfer, which generates two transconjugants per conjugative pairing (*19*).

### A new method for conjugation quantification

In the absence of growth dynamics, the conjugation efficiency (η), or the rate constant of conjugation, can be determined as

η=T/(DRΔt)

where *D*, *R*, and *T*, respectively, represent the densities of the donor, recipient, and transconjugant populations after a short incubation time (Δ*t*) (*9*). This relationship can be disrupted via selection (e.g., media and antibiotics) that leads to differential growth or death (Fig. 1A). To avoid these confounding factors, we restrict growth during conjugation (fig. S1). Under these conditions, *D* and *R* can be readily determined from starting cultures, which poses no technical challenges.

**Fig. 2:**
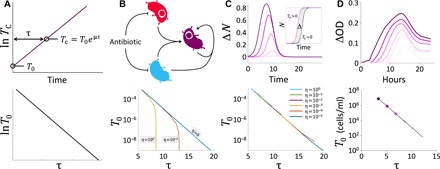
A time to threshold method for conjugation quantification. (**A**) The principle of time to threshold quantification. Consider the exponential growth of the transconjugant from an initial density *T*_0_ (top panel). The time (τ) required for the population to reach a set threshold (*T_C_*) is uniquely determined by *T*_0_ and the specific growth rate (μ). This defines a log-linear relationship between *T*_0_ and τ: ln*T*_0_ = ln *T*_C_ − μτ (bottom panel). (**B**) Quantification of *T*_0_ is complicated by the presence of donor and recipient cells. Top panel: Although strong antibiotic selection is applied against donor and recipient cells during transconjugant outgrowth, death is not instantaneous (i.e., conjugation may still occur). Bottom panel: Modeling reveals conjugation of variable efficiency (η) during outgrowth causes a deviation from the log-linear relationship. This effect is amplified with smaller *T*_0_, where transconjugants produced from outgrowth conjugation—not outgrowth alone—may comprise a sizeable proportion of the total transconjugant population. (**C**) Correcting for outgrowth conjugation. Top panel: The growth contribution from the transconjugant alone can be approximated by the difference (Δ*N*) between the growth curves originating from the conjugation mixture (*T*_0_ > 0) and conjugation control (*T*_0_ = 0). Darker curves represent higher *T*_0_. Bottom panel: Using Δ*N*, the log-linear relationship between *T*_0_ and τ is maintained even in the presence of conjugation during outgrowth. (**D**) Applying the time to threshold method to experimental data. *T*_0_, spanning six orders of magnitude, maintains a strong correlation (*R*^2^ > 0.99) with τ from a ΔOD threshold. Darker curves represent higher *T*_0_.

Quantifying *T* after incubation traditionally relies on selective plating from mixed culture, which is error prone and tedious. Relatively small *T* and variation over multiple orders of magnitude make this more challenging, and restricting growth exacerbates these issues. To overcome these limitations, we developed a simple, yet robust method that allows transconjugant outgrowth for improved quantification. Consider the exponential growth of the transconjugant starting from an initial density of *T*_0_. *T*_0_ is uniquely related to τ, the time for the population to reach a set threshold *T*_C_

$$\text{ln}T_{0}=\text{ln}T_{C}-\mu \tau$$

where μ is the specific growth rate (Fig. 2A). With proper calibration, this relationship provides a high-throughput approach to quantify *T*_0_ by tracking bacterial growth via optical density (OD) in a plate reader. Experimentally, we establish a standard curve relating *T*_0_ and τ by growing transconjugant cultures from known starting densities. The standard curve is then used to determine *T*_0_ in samples subjected to the same growth conditions. For the same *T*_C_, the standard curves for different transconjugants (generated from different donors, but the same recipient) were highly similar to each other, reflecting a similar μ for these strains (fig. S2A). For the same transconjugant, we found quantification to be robust to the choice of OD thresholds that fall in the logarithmic growth phase (fig. S2B).

The reliability of the standard curve depends on a critical assumption: All the growth originates from the transconjugants at time zero. This can be approximated by imposing strong double selection to suppress growth of donor and recipient cells during outgrowth. However, this suppression is not instantaneous, and new transconjugants can still be produced through conjugation (Fig. 2B).

To examine this contribution, we built a kinetic model that accounts for growth and conjugation dynamics during *T* outgrowth. In the absence of conjugation during outgrowth (η = 0), the model predicts a linear relationship between ln*T*_0_ and τ, similar to the case where exponential growth is assumed (Fig. 2A). At η > 0, the correlation deviates from the log-linear relationship, where the same *T*_0_ would correspond to a smaller τ than what would be expected for η = 0. A larger η leads to a larger deviation. Under this condition, a given value of τ would lead to an overestimate of *T*_0_ if the log-linear relationship is directly used. However, we found that the deviation from outgrowth conjugation can be eliminated by subtracting the growth curve produced by parents mixed only at the start of outgrowth, yielding Δ*N* value (Fig. 2C). The early phase of Δ*N* provides an approximation of *T*_0_ growth in the absence of outgrowth conjugation, and simulated standard curves established using Δ*N* do not significantly deviate from the log-linear correlation even in the presence of substantial outgrowth conjugation. Experimental results using OD to approximate *N* emulate the model, and τ displays a strong log-linear correlation with *T*_0_ over multiple orders of magnitude (Fig. 2D and fig. S2).

### Antibiotic modulation of conjugation

**Fig. 3:**
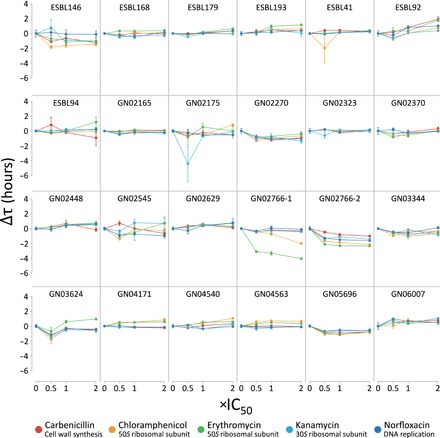
Antibiotic modulation of conjugation is rare in pathogenic *E. coli.* The effect of five antibiotics on conjugation in clinical *E. coli* pathogens was assessed via the time to threshold method. Antibiotics of differing therapeutic mechanism were dosed in three concentrations (0.5×, 1×, and 2×) based on 50% inhibitory concentrations for a MG1655 *E. coli* recipient standard. IC_50_ values for Carb, Cm, Kan, erythromycin (Ery), and Norf were as follows: 1.91, 1.92, 2.13, 20.20, and 0.05 μg/ml. Displayed Δτ are averages of triplicate measurements ±SE, and normalized by subtracting the no-antibiotic control τ. Promotion of conjugation is indicated by Δτ < 0, while Δτ > 0 indicates inhibition. Only GN02766 displayed major modulation of conjugation when exposed to Ery (all concentrations, *P* < 0.01, Tukey post hoc test) and Cm (2× concentration, *P* < 0.05). Results from two separate GN02766 experiments are shown.

Together with seven previously identified ESBL donors (*20*), we measured the effect of antibiotics on conjugation in clinical *E. coli* pathogens (Fig. 3). We display the difference in time to threshold (Δτ) between antibiotic and no-antibiotic control conditions to better reflect conjugation effects across isolates and minimize unnecessary data processing. The absolute magnitude of τ is the result of many variables that affect growth rate (e.g., conjugation and media conditions). Five antibiotics with different mechanisms of action were tested, covering inhibition of translation, DNA replication, and cell wall synthesis. Three sublethal concentrations were used, based on the 50% inhibitory concentrations (IC_50_) of the susceptible recipients, to capture concentration-dependent effects (*9*). Donor minimum inhibitory concentrations (MICs) and IC_50_s are shown in fig. S3A and table S3. We erred on the side of concentrations too low for the multidrug-resistant pathogens to ensure the survival of the recipients and subsequent transconjugants. Because of experimental constraints chosen to restrict background conjugation and growth dynamics, donors with conjugation efficiencies less than 10^−16^ produced too few transconjugants for quantification.

A global analysis of variance (ANOVA) revealed significant effects for both antibiotic × isolate [*F*(94,1374) = 4.96, *P* < 0.0001] and antibiotic × concentration × isolate [*F*(282,999) = 2.28, *P* < 0.0001] interactions, prompting post hoc testing via Tukey post hoc test. We find that the majority of pathogen donors display little to no significant antibiotic modulation of conjugation, and where there is statistically significant Δτ, it corresponds to approximately less than a fivefold change in transconjugants. In addition, no correlation was seen between donor IC_50_ and Δτ (fig. S3B). However, isolate GN02766 exhibited a drastic increase in conjugation when exposed to certain antibiotics. GN02766 produced ~3.5-fold more transconjugants in the presence of 2× IC_50_ of Cm [*t*(7.5) = 3.7, *P* < 0.05, Welch’s modified *t* test], while all concentrations of erythromycin (Ery) yielded up to 31-fold more transconjugants [*t*(8.5) = 18, *P* < 0.0001] (fig. S4A). Of the five antibiotics tested, only Cm and Ery are considered bacteriostatic and both inhibit translation via the 50*S* ribosomal subunit, suggesting a common mechanism. Kan, which also inhibits translation, did not have an effect, possibly due to bactericidal effects, targeting the 30*S* ribosomal subunit, or concentration dependence.

To distinguish a general bacteriostatic effect from a macrolide- and phenicol-specific mechanism, we retested GN02766 with azithromycin (Az) and sulfamethoxazole (Sm). Solvent- and donor-recipient strain interactions were also tested and ruled out (fig. S4B). Also a macrolide, Az differs from Ery in its 15-membered ring structure. Sm is bacteriostatic, but differs from Cm and Ery in mechanism of action by inhibiting folate and, subsequently, DNA synthesis. Az produced a virtually identical ~31-fold increase in transconjugants to Ery [*t*(8.4) = 15.5, *P* < 0.0001], while Sm produced no significant effect [*t*(2) = −0.04, *P* = 0.76] (fig. S4C). Together, these results suggest macrolide- and, to a lesser degree, phenicol-specific promotion of conjugation in GN02766.

### Conjugation phenotype to plasmid genotype

To reveal plasmid genotype and identify features associated with antibiotic modulation and conjugation efficiency, we performed whole-genome sequencing on 19 isolates of varying conjugative phenotypes (BioProject no. PRJNA551684). For large plasmids, standard plasmid purification kits yielded poor results, and sequence assembly from short reads has been shown to be difficult. Instead, we used long-read sequencing from Pacific Biosciences (PacBio) capable of accurately discerning mobile elements from the chromosome. As predicted from empirical findings, plasmid-borne β-lactamases were detected in each isolate. A summary of sequencing results can be found in table S2, complete with plasmid number, size, replicon (Inc), mobility relaxase (MOB), and identified resistances present in each isolate.

**Fig. 4:**
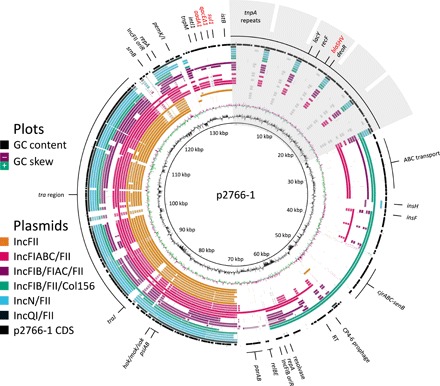
Identification of plasmid features unique to strain GN02766. Plasmid sequences from strains displaying no antibiotic modulation were aligned to p2766-1 via BLASTn and BLAST Ring Image Generator (BRIG) (*47*). Nucleotide identity ≥70% is indicated by a band colored according to the Inc group, with darker shading corresponding to higher sequence match. Blank regions indicate <70% nucleotide identity. Inner GC content plots, size map, and outer coding sequences (CDSs) are for p2766-1. Antibiotic resistance genes are highlighted in red, and transposon repeat region in gray. Notable features include five-plasmid maintenance systems (*pemKI*, *relBE*, *hok-sok*, *srnBC*, and *parAB*), a mobilized enterotoxin and colicin J receptor operon (*cjrABC-senB*), and five consecutive *tnpA* transposon repeats carrying *blaSHV*.

GN02766 was found to harbor two plasmids: p2766-1, a 137-kb plasmid identified as IncFIB/IncFII/Col156, and a 29-kb plasmid without designation. Without identifiable MOB or transfer (*tra*) machinery on the 29-kb plasmid, only p2766-1 appears to be mobile and carries a β-lactamase, *blaSHV*, relevant to our conjugation assay. By comparing the plasmids of isolates displaying no antibiotic modulation to p2766-1, we can identify unique features that may give insight into how macrolide and phenicol antibiotics promote conjugation. Here, we show 24 of these plasmids that bear the greatest similarity to p2766-1 (nucleotide identity, ≥70%), although much diversity remains (Fig. 4). Much of the *tra* region for conjugation was conserved across plasmids with *traJ*, a key activator of *tra* gene expression, as a notable exception. We also aligned plasmid sequences to p168-1, the closest match to p2766-1 that displayed no antibiotic modulation, to see what common features p2766-1 might lack (fig. S5). No major commonalities among the majority of plasmids appear to be lost, suggesting the conjugation phenotype of p2766-1 may be gained instead.

There are several features of note on p2766-1: five plasmid maintenance systems, a mobilized enterotoxin and colicin J receptor operon, and five consecutive *tnpA* transposon repeats. Of the five plasmid maintenance systems, four function through addiction (*pemKI*, *relBE*, *hok-sok*, and *srnBC*), with toxins that kill daughter cells if they lack the plasmid-encoded antitoxin. For comparison, ESBL-producing strains only have 2.38 addiction systems on average with a range of 0 to 6 (*21*). The fifth maintenance system, *parAB*, aids in plasmid segregation (*22*). In common with two other IncFIB/FII/Col156 plasmids, p2766-1 also includes *cjrABC* and *senB*, which play important roles in *E. coli* pathogenesis (*23*). Unique to p2766-1 is a repetitive region of five consecutive *tnpA* transposon repeats, each bearing a *deoR*, *blaSHV*, *recF*, and truncated *lacY* gene, respectively, encoding a transcriptional repressor of sugar metabolism (*24*), ESBL, DNA recombination and repair protein, and β-galactoside permease. This repetitive region is genuine as we see no decrease in sequence coverage indicative of assembly error (fig. S6). It is particularly interesting that *deoR* is implicated in limiting plasmid copy number (*25*), and *recF* facilitates recombination (*26*, *27*) and mutagenesis (*28*). A closely related IS26-flanked *bla*_SHV-5_ amplicon was found to spread via recombination, not transposition, with up to 11 consecutive amplicons forming under high β-lactam antibiotic stress (*29*).

The conjugation phenotypes we observed with clinical *E. coli* pathogens can arise from the plasmids, chromosomes, or both. To control for chromosomal effects, we used Kan-susceptible DA28102 transconjugants (denoted as strain #T) as donors to Kan-resistant fAYC002 recipients. With plasmids as the only differences between the transconjugants, any change in conjugation efficiency may therefore be attributable to the plasmids themselves. In this way, the macrolide promotion seen in GN02766 appears to be transferrable, with 0.5× and 1× IC_50_ of Ery significantly reducing τ (*P* < 0.0001 and *P* < 0.05, respectively, Tukey post hoc test) when 2766T is used as a donor (fig. S7A). No effect was seen with other DA28102 transconjugants, further supporting a p2766-1–based mechanism (fig. S7B). Donor, recipient, and growth conditions likely play a role, however, as the degree of macrolide promotion was diminished in 2766T.

### Genetic markers of conjugation efficiency

Compared to the maximum 31-fold change we saw with antibiotics, the baseline conjugation efficiencies of plasmids have the potential to differ over several orders of magnitude. Even relatively minor changes, such as growth stage or nutrient concentration, can cause orders of magnitude shifts in conjugation efficiency (*9*, *10*, *30*). Here, we focus on the origin of these more fundamental distinctions between conjugative plasmids. Plasmids are typically classified by two major systems: Inc group and MOB relaxase. Inc groups are based around plasmid maintenance (e.g., replication controls), where two plasmids of the same Inc group could not be stably maintained in the same cell (*31*). MOB relaxase typing, centered on the protein that nicks *oriT* to initiate conjugative transfer, was proposed to avoid problems in the Inc system, such as multiple replicons or maintenance systems that differ greatly in how they work (*32*).

**Fig. 5:**
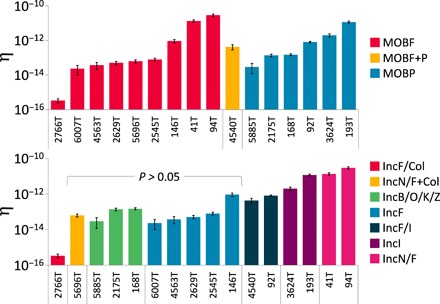
Conjugation efficiency groups by plasmid incompatibility. Plasmids carrying β-lactamases from clinical *E. coli* pathogens were classified by Inc grouping and MOB relaxase families. To eliminate host effects, conjugation efficiencies were only measured using DA28102 transconjugants and the fAYC002 recipient. There is no significant difference among conjugation efficiencies when grouped by MOB relaxase (*P* = 0.291, ANOVA), whereas grouping by Inc is highly significant (*P* < 0.0001). Individual Inc groups that could not be distinguished from one another at *P* ≤ 0.05 are bracketed. Data were log transformed to normalize variation across orders of magnitude. Error bars represent ±SD from at least three replicates.

MOB relaxases are thought to accurately represent *tra* machinery based on high congruence between their phylogenetic trees (*32*). Therefore, we wondered whether MOB relaxase might correlate with conjugation efficiency with the donor, recipient, and environment held constant. However, we find that Inc groups are more predictive of a plasmid’s conjugation efficiency than MOB relaxase [*F*(6,53) = 111.6, *P* < 0.0001 versus *F*(2,57) = 1.26, *P* = 0.291, respectively, ANOVA; Fig. 5). The strong correlation between the Inc group and conjugation efficiency remains when the 2766T outlier is removed [*F*(5,48) = 62.36, *P* < 0.0001]. Inc groupings were made at the most general level (i.e., IncF includes IncFI, FII, etc.) for plasmids carrying a β-lactamase marker by which we measured conjugation. Given the apparent difference between the IncF/Col and IncF groups, we paid particular attention to mobilizable colicin plasmids that can transfer alongside the β-lactamase (i.e., from GN05696 to 5696T and beyond). Further subdivisions in Inc groups and MOB types may yield greater resolution between conjugation efficiencies. Our results suggest that the factors involved in plasmid maintenance play a more significant role in its transfer than previously thought.

## DISCUSSION

Drawing from the same fundamental principles as quantitative polymerase chain reaction, we developed time to threshold as a simple and effective new method for quantifying plasmid transfer rates in the absence of confounding growth dynamics. The time to threshold method improves upon agar plating in both throughput and sensitivity, returning transconjugant density in as little as 5 hours without the ambiguity from subjective colony counting. Amid conflicting reports for antibiotic modulation of conjugation (*9*, *11*, *15*, *16*), we find little to no effect in this largest study of conjugation phenotype in clinical *E. coli* pathogens. The lack of a general antibiotic effect suggests that modulation, if present, arises from more specific interactions and highlights the potentially confounding impact of growth dynamics on conjugation quantification. Yet, even minor modulation of conjugation maintained over time may be sufficient to push plasmids above or below critical maintenance thresholds (*7*). As more conjugation inhibitors and promoters are discovered, we may begin to harness and incorporate these dynamics into prophylactic strategies that address the source of antibiotic resistance for many pathogens (*5*).

Isolate GN02766 is an exceptional case of conjugation modulation with the ability to spread antibiotic resistance faster in the presence of antibiotics. Through the time to threshold method, we detect up to 31-fold promotion of conjugation upon exposing GN02766 to macrolides or Cm, both of which target the 50*S* ribosomal subunit. We link macrolide promotion to a single IncFIB/FII/Col156 plasmid (i.e., p2766-1) bearing five plasmid maintenance systems and *tnpA* transposon repeats notably encoding *deoR*, *blaSHV*, and *recF*. Unique to p2766-1 and the conjugation promotion phenotype, *deoR* and *recF* are involved in suppressing copy number (*25*) and promoting recombination of plasmids, respectively. Translation inhibition from macrolides and Cm may relieve metabolic suppression from multiple copies of *deoR* and destabilizing oligomerization from *recF* (*33*), increasing p2766-1 copy number, *tra* gene expression, and, ultimately, conjugation efficiency. As the number of unique β-lactamases continues to rise, the potential this transposon offers for *blaSHV* diversification through gene duplication is concerning (*29*). Both intra- and intercellularly mobile through recombination and antibiotic-promoted conjugation, this *blaSHV* transposon represents a threat for the evolution of antibiotic resistance and warrants further study.

Going beyond special cases, understanding the determinants underlying plasmid mobility is critical to predicting how they spread as vehicles of antibiotic resistance and HGT. By removing host and growth variables, we find that Inc groups based on plasmid maintenance appear predictive of conjugation efficiency, whereas MOB relaxase classification does not. This comes as a surprise given MOB relaxases’ more direct connection to conjugation (*32*). However, the dominance of MOBF and MOBP among *E. coli* isolates with detectable conjugation falls in stark contrast to the diversity of Inc groups, suggesting that MOB may dictate plasmid prevalence at a high level (*31*, *34*). The apparent predictive power of incompatibility groups, as ubiquitous features of plasmids, could nevertheless hold considerable value for understanding gene flow through HGT networks, especially if it extends beyond *E. coli* and the plasmids studied herein. Knowing which plasmids and bacterial strains are most adept at mobilizing antibiotic resistance could better guide strategies to inhibit its spread and improve the life span of antibiotics (*5*, *7*, *35*).

## MATERIALS AND METHODS

### Bacterial strains

A full description of all strains used in this study can be found in table S1.

### Growth media

Unless otherwise stated, donor or recipient strains were cultured at 37°C in standard Luria-Bertani (LB; Miller) broth with shaking. Conjugation experiments were performed in M9 media containing casamino acid (2 mg/ml), thiamine (0.1 mg/ml), 2 mM MgSO_4_, 0.1 mM CaCl_2_, and 0.4% (w/v) glucose. We call this M9CA media in subsequent text. Rich LB or terrific broth (TB) was used to generate high cell density for subsequent conjugation in M9CA, which was used to control for growth dynamics. Shaking is applied for oxygenation, accurate OD measurements, and breaking up conjugation pili.

### Antibiotic resistance screening

We screened the pathogen library for Carb, Cm, and Kan resistance to establish selection markers for conjugation quantification. The BSIB had previously collected disk diffusion data on β-lactam antibiotics other than Carb, which we used to infer Carb susceptibility (CarbS) for 51 isolates. Isolates without prior data or with ambiguous resistance were inoculated in 1 ml of LB media in 96–deep well plates, covered in a gas-permeable membrane, and grown overnight. Once grown, cultures were diluted 1000× into secondary 96-well plates with and without added antibiotic. Concentrations for antibiotics were as follows: Carb (100 μg/ml), Cm (50 μg/ml), and Kan (50 μg/ml). Secondary plates were then grown for approximately 16 hours. At this point, the OD_600_ of each culture was measured in a plate reader. Antibiotic culture OD was normalized to corresponding no-antibiotic cultures. Resistance to each antibiotic was defined as ≥1% maximal growth.

The MIC, which we defined as the first antibiotic concentration to yield 10% or less of the no-antibiotic control’s maximum OD_600_, and IC_50_ were also determined for plasmid donors tested for antibiotic effects on conjugation (fig. S3A and table S3). Plasmid donors were grown for 16 hours, diluted 100-fold into M9CA with antibiotic concentrations ranging from 0 to 64 μg/ml, and then grown for 24 hours before taking endpoint OD_600_ measurements.

### Conjugation screening

To find pathogens capable of conjugation, we screened Carb-resistant isolates for the ability to transfer β-lactamase into Carb-susceptible recipients. Pathogen cultures were grown overnight in 1 ml of LB media with Carb (100 μg/ml) in 96–deep well plates covered in a gas-permeable membrane. Recipients were grown overnight in 3 ml of LB media with either Kan (50 μg/ml) or Cm (70 μg/ml). Pathogen isolates were then diluted 10× in LB media and regrown under similar conditions for 2 hours to enter exponential phase. Exponential-phase pathogen isolates and recipient were diluted 100× into 200 μl of LB media and mixed 1:1 in a 96-well plate with dual antibiotic selection for transconjugants. Mixed cultures were then covered with 50 μl of mineral oil and grown in a plate reader for 24 hours.

After 24 hours of exposure to transconjugant selection, mixed pathogen and recipient cultures that displayed growth were diluted 1000× into fresh LB media with antibiotic selection for transconjugants. These cultures were covered with 50 μl of mineral oil and regrown in a plate reader for 24 hours. Cultures that repeated growth under high dilution and strong transconjugant selection were plated on agar also selecting for transconjugants. Individual colonies were isolated and grown overnight in 3 ml of LB media with transconjugant selection. These clonal transconjugant cultures were glycerol stocked for later analysis.

### Transconjugant quantification via time to threshold

Donor *E. coli* strains were inoculated into 3 ml of TB media with Carb (100 μg/ml) and grown for approximately 16 hours at 37°C without shaking. An appropriate recipient MG1655 *E. coli* strain (CmR DA28102 or KanR fAYC002) with contrasting resistance markers was similarly grown, but with 50 μg/ml selecting antibiotic and shaking. Following growth, donor cultures were diluted 10× and regrown for 2 hours to enter the exponential phase. Recipient and exponential-phase donor cultures were pelleted at 2000 relative centrifugal force (rcf) for 10 min at 25°C and resuspended in M9CA media, equalizing OD_600_. At this point, aliquots of donor and recipient cultures were taken for quantification via agar plating and controls for outgrowth conjugation.

Donor and recipient cultures were then mixed in a 1:1 ratio for conjugation, distributed in 500-μl volumes, and incubated for 1 hour at room temperature while being exposed to antibiotic or control test conditions. Antibiotic test conditions were applied in 0.5×, 1×, and 2× multiples of IC_50_s determined for the primary MG1655 *E. coli* recipient. IC_50_ values for Carb, Cm, Kan, Ery, and Norf were as follows: 1.91, 1.92, 2.13, 20.20, and 0.05 μg/ml (*9*). These growth conditions were chosen to restrict growth during conjugation. After 1 hour, cell mixtures were vortexed to disrupt conjugation, pelleted, and resuspended in 500 μl of M9CA. The separated donor and recipient conjugation control aliquots were then mixed to track outgrowth conjugation.

The experimental and outgrowth conjugation control curves must be displaced in time; otherwise, ΔOD will not reach target OD_600_ thresholds. For this reason, all cell mixtures were diluted 150× in M9CA with dual antibiotic selection [Carb (100 μg/ml), Cm (70 μg/ml), and Kan (50 μg/ml)] according to the donor-recipient pairs and then distributed three times in 150-μl volumes onto a microtiter plate. Microtiter cultures were covered with 50 μl of mineral oil to prevent evaporation and grown in a plate reader for 24 hours at 37°C, shaking before each OD_600_ measurement. The 150× dilution was chosen on the basis of an estimated η of ~10^−14^ from previous growth-restricted *E. coli* measurements. For best results, *T*_0_ should be maximized while minimizing outgrowth η. Outgrowth dilutions and temperature may need to be adjusted on a strain-by-strain basis to optimize separation between experimental and control curves. For *E. coli*, the most common plasmids belong to the IncF family. Therefore, to maximize *T*_0_ with the *E. coli* library, we used rich, buffered TB media for optimal conjugative pili formation and exponential-phase donor cultures for activation of F plasmid transfer machinery (*30*). Conversely, recipients are kept in stationary phase for decreased motility or cell wall modifications thought to improve pili tip searching (*9*, *36*).

Transconjugant growth curves were passed through a moving average filter in MATLAB to reduce noise. Time to specified OD_600_ threshold was found via linear interpolation. OD_600_ thresholds were typically chosen between 0.03 and 0.05 in early exponential phase to reduce background noise, maintain the cell density:OD relationship, and lessen postantibiotic effects (*37*). Within this range, changes in OD threshold had little effect on the correlation between τ and T_0_ (fig. S2B).

### Standard curves

Transconjugant cultures were grown following the time to threshold protocol to accurately recreate postconjugation outgrowth conditions. Once resuspended in M9 media with Carb (100 μg/ml) and Cm (70 μg/ml), the cultures were serially diluted by 10^7^ and plated on LB agar to determine *T*_0_ via colony-forming units (CFUs). These dilutions were then covered in 50 μl of mineral oil and grown in a plate reader as before for 24 hours. With known *T*_0_, we constructed standard curves for each transconjugant strain by calculating τ from growth curves at varied OD thresholds. We find that defined M9 with Carb (100 μg/ml) and Cm (70 μg/ml) antibiotic concentrations minimizes standard curve variation across strains.

### Statistical analysis

Time to threshold results (τ) were normalized by subtracting τ from the no-antibiotic control yielding a Δτ value. An outlier for both ESBL41 and ESBL168 was removed due to unambiguous plate reader and experimental error, respectively, to preserve data integrity. Triplicate τ measures were parsed with ANOVA in *R* to reveal significant global interactions among strain, antibiotic, and concentration variables. Tukey post hoc test was performed for significant interactions. All tests are two tailed, and all replicates are technical unless indicated otherwise.

### Genome sequencing and assembly

We selected 19 strains for whole-genome sequencing covering a range of conjugation dynamics. Genomic DNA was extracted using the QIAGEN MagAttract high–molecular weight DNA kit (catalog ID: 67563) from cultures grown for 16 hours until a density of approximately 10^9^ cells/ml. DNA libraries were prepared according to PacBio’s recommendations and sequenced using the PacBio Sequel system.

All samples were assembled and polished using the PacBio SMRT Analysis assembly pipeline (SMRTLink version 5.1.0.26412), which uses HGAP 4 for assembly and Quiver for assembly polishing. Default parameters were applied, with the following exceptions: genome length was set to 5 Mb, the aggressive option was set to true, no read quality filters were applied, and the default falcon configuration parameters were supplanted with the following parameters: pa_DBsplit_option = -x500 -s200; ovlp_DBsplit_option = -x500 -s200. For samples with initial suboptimal assemblies, seed coverage was modified to increase the proportion of corrected reads. Seed coverage was set to 60× for ESBI168, GNO2448, and GNO4540, and 35× for GNO2766.

### Sequence analysis

For constructing the phylogenetic tree, single-nucleotide polymorphisms (SNPs) were identified from genome assemblies (BioProject accession nos. PRJNA290784 and PRJNA551684) following the North Arizona SNP Pipeline (NASP) pipeline (*38*) using *E. coli* strain EC958 (GenBank accession no. HG941718.1) as a reference. Duplicated regions of the reference genome, including repeat regions and multiple gene copies, were determined by aligning the reference sequence to itself using the NUCmer-3.23 (*39*). SNPs that fell within these duplicate regions were excluded from further analysis to avoid false SNP calls due to ambiguous read alignment. Each query genome assembly was aligned to the reference with NUCmer-3.23. The best SNPs in all genomes compared to the reference were concatenated in a matrix. Phylogenetic trees were inferred using the maximum likelihood (ML) method available in RAxML (*40*, *41*). This returned the best-found tree from 100 replicates inferred using the general time-reversible substitution model.

For Inc typing, the plasmid sequences were queried against a locally downloaded version (retrieved 22 May 2018) of the PlasmidFinder database (http://www.genomicepidemiology.org), using the recommended percentage coverage threshold of 60% and a more stringent percentage identity and e-value thresholds of 90% and 0.00001, respectively (*42*). MOB typing was performed as previously described, and default value was chosen for all parameters (*43*). Specifically, the e-value threshold of each MOB type was chosen as follows: MOBC, 0.001; MOBF, 0.01; MOBH, 0.01; MOBP, 1; MOBQ, 0.0001; and MOBV, 0.01. In addition, 247,882 protein sequences were extracted from a National Center for Biotechnology Information dataset of 6952 complete Enterobacteriaceae plasmids (*44*) and were compiled into a database in combination with the sequences of 2423 antibiotic resistance proteins from ResFinder database (retrieved 22 May 2018) (http://www.genomicepidemiology.org) (*45*) as predicted by prodigal v2.6.3 (*46*). Genome assemblies were further annotated using Prokka v1.13 (*47*) against the above compiled protein database. For comparison, plasmids were aligned to one another via BRIG, displaying sequence matches with nucleotide identity ≥70% (*48*).

### Transconjugant conjugation efficiency

Transconjugant conjugation was performed similarly to the time to threshold protocol with a few exceptions. Transconjugant donors were grown with shaking as DA28102 displays poor growth otherwise and fAYC002 was the sole recipient used. Donor, recipient, and second-generation transconjugant cell densities were quantified through LB agar plating and CFU counting.

## Supplementary Material

http://advances.sciencemag.org/cgi/content/full/6/4/eaax3173/DC1

Download PDF

Environmental and genetic determinants of plasmid mobility in pathogenic Escherichia coli

## SUPPLEMENTARY MATERIALS

Supplementary material for this article is available at http://advances.sciencemag.org/cgi/content/full/6/4/eaax3173/DC1 Fig. S1. Conditions during the conjugation period effectively decouple conjugation from growth dynamics. Fig. S2. The log-linear correlation between *T*_0_ and τ is maintained with respect to strain and choice of OD threshold. Fig. S3. Antibiotic resistance of plasmid donors. Fig. S4. Antibiotic modulation of conjugation for strain GN02766. Fig. S5. Identification of plasmid features lost in strain GN02766. Fig. S6. Sequencing read coverage plot for p2766-1. Fig. S7. Macrolide promotion of conjugation is transferrable. Table S1. All strains used in this study. Table S2. Plasmid composition of pathogenic *E. coli* isolates. Table S3. MICs and IC_50_s for plasmid donors in this study. Model development and assumptions Reference (*49*)

## Appendix

View/request a protocol for this paper from *Bio-protocol*.
