# Supplementary material for: Environmental and genetic determinants of plasmid mobility in pathogenic Escherichia coli
Source: Sci Adv. 2020 Jan 24;6(4):eaax3173. doi: 10.1126/sciadv.aax3173 (PMC6981087; doi:10.1126/sciadv.aax3173)
Supplement: http://advances.sciencemag.org/cgi/content/full/6/4/eaax3173/DC1 [file supp_6_4_eaax3173__index.html]

Science Advances | Science AdvancesAAASSearchScience AdvancesMenu

## Supplementary Materials

**This PDF file includes:**

- Fig. S1. Conditions during the conjugation period effectively decouple conjugation from growth dynamics.
- Fig. S2. The log-linear correlation between *T*0 and τ is maintained with respect to strain and choice of OD threshold.
- Fig. S3. Antibiotic resistance of plasmid donors.
- Fig. S4. Antibiotic modulation of conjugation for strain GN02766.
- Fig. S5. Identification of plasmid features lost in strain GN02766.
- Fig. S6. Sequencing read coverage plot for p2766-1.
- Fig. S7. Macrolide promotion of conjugation is transferrable.
- Table S1. All strains used in this study.
- Table S2. Plasmid composition of pathogenic *E. coli* isolates.
- Table S3. MICs and IC50s for plasmid donors in this study.
- Model development and assumptions
- Reference (*49*)

Download PDF

**Files in this Data Supplement:**

- Adobe PDF - aax3173\_SM.pdf
