# Supplementary material for: Environmental and genetic determinants of plasmid mobility in pathogenic Escherichia coli
Source: Sci Adv. 2020 Jan 24;6(4):eaax3173. doi: 10.1126/sciadv.aax3173 (PMC6981087; doi:10.1126/sciadv.aax3173)
Supplement: Download PDF [file aax3173_SM.pdf]

Science Advances
AAAS

advances.sciencemag.org/cgi/content/full/6/4/eaax3173/DC1

Supplementary Materials for

Environmental and genetic determinants of plasmid mobility in pathogenic *Escherichia coli*

Jonathan H. Bethke, Adam Davidovich, Li Cheng, Allison J. Lopatkin, Wenchen Song, Joshua T. Thaden, Vance G. Fowler Jr., Minfeng Xiao, Lingchong You*

*Corresponding author. Email: you@duke.edu

Published 24 January 2020, *Sci. Adv.* **6**, eaax3173 (2020)
DOI: 10.1126/sciadv.aax3173

This PDF file includes:

Fig. S1. Conditions during the conjugation period effectively decouple conjugation from growth dynamics.
Fig. S2. The log-linear correlation between T_0 and τ is maintained with respect to strain and choice of OD threshold.
Fig. S3. Antibiotic resistance of plasmid donors.
Fig. S4. Antibiotic modulation of conjugation for strain GN02766.
Fig. S5. Identification of plasmid features lost in strain GN02766.
Fig. S6. Sequencing read coverage plot for p2766-1.
Fig. S7. Macrolide promotion of conjugation is transferrable.
Table S1. All strains used in this study.
Table S2. Plasmid composition of pathogenic *E. coli* isolates.
Table S3. MICs and IC_{50}s for plasmid donors in this study.
Model development and assumptions
Reference (*49*)

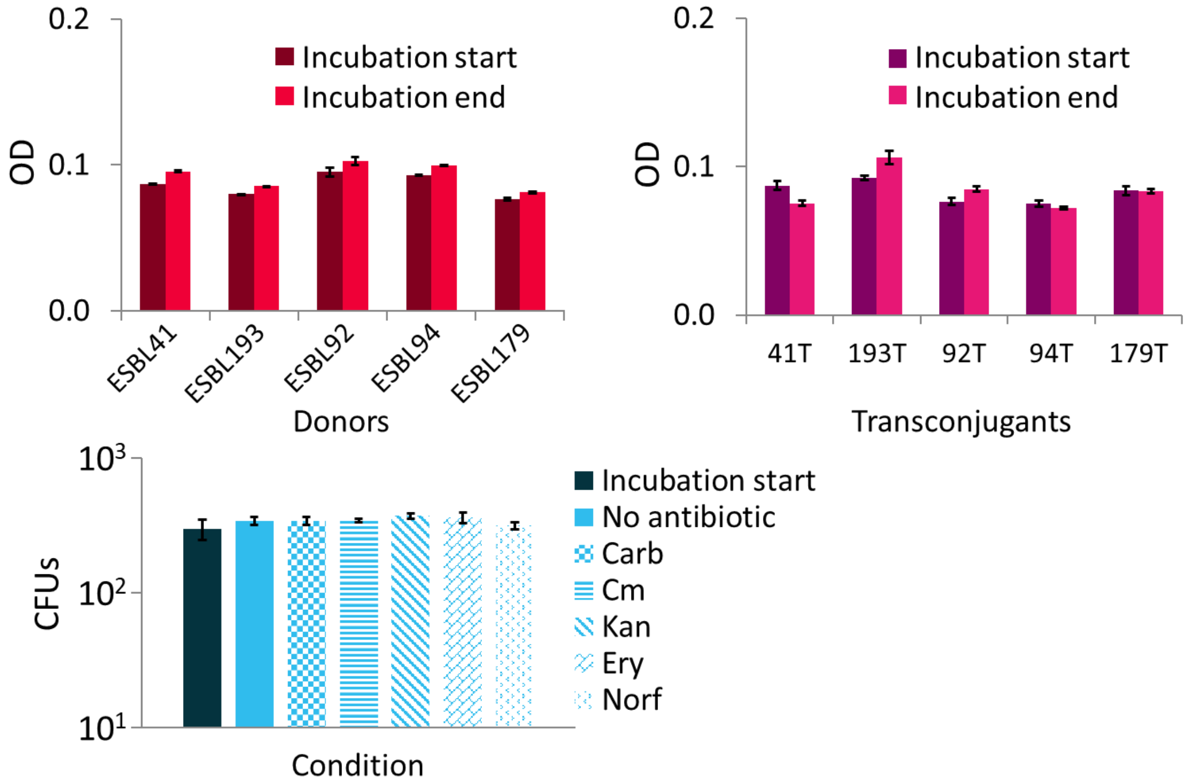

Fig. S1. Conditions during the conjugation period effectively decouple conjugation from growth dynamics. (**a**) The OD_{600} of five *E. coli* pathogen donors changes minimally in M9CA media at room temperature (25°C) over 1 hour. Error bars represent +/- SE from triplicate measurements. (**b**) The OD_{600} of five *E. coli* transconjugants changes minimally in M9CA media at room temperature (25°C) over 1 hour. Error bars represent +/- SE from triplicate measurements. (**c**) MG1655 recipient cell density did not significantly change under conjugation growth conditions, with or without added antibiotic. Antibiotics were added at the highest concentration used during conjugation (2x IC_{50}). Error bars represent +/- SE from triplicate measurements.

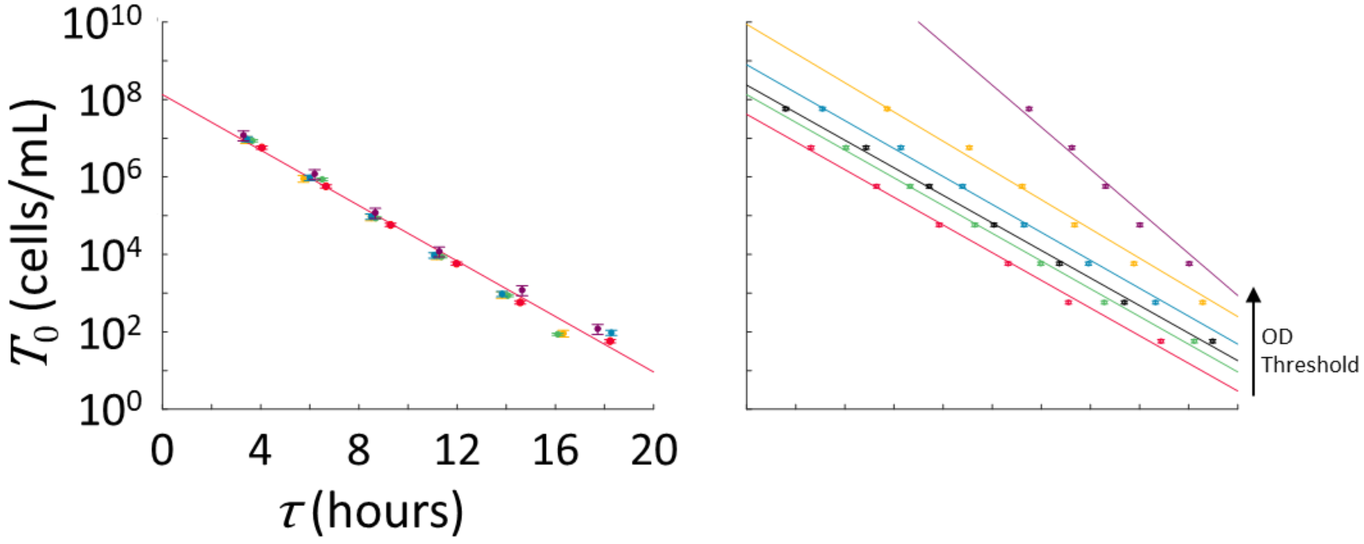

Fig. S2. The log-linear correlation between T_0 and τ is maintained with respect to strain and choice of OD threshold. (**a**) With a constant OD_{600} threshold of 0.03, the standard curves for ESBL193, ESBL168, GN02545, GN02766, and GN05696 display only minor variations in the relationship between T_0 and τ. For example, data and curve for ESBL193 are shown in red, with other strains' data alternatively colored. Serially diluted transconjugants were grown under conditions recreating post-conjugation outgrowth to establish the relationship between T_0 and τ. Error bars represent +/- SD from triplicate measurements. (**b**) Standard curves are for ESBL193 with changing OD_{600} thresholds of 0.01, 0.03, 0.05, 0.1, 0.2, and 0.3 (bottom to top). Serially diluted transconjugants were grown under conditions recreating post-conjugation outgrowth to establish the relationship between T_0 and τ. As OD_{600} threshold increases, τ also increases and shifts the curve. The relationship between T_0 and τ degrades as cell growth slows near the end of exponential phase. Error bars represent +/- SD from triplicate measurements.

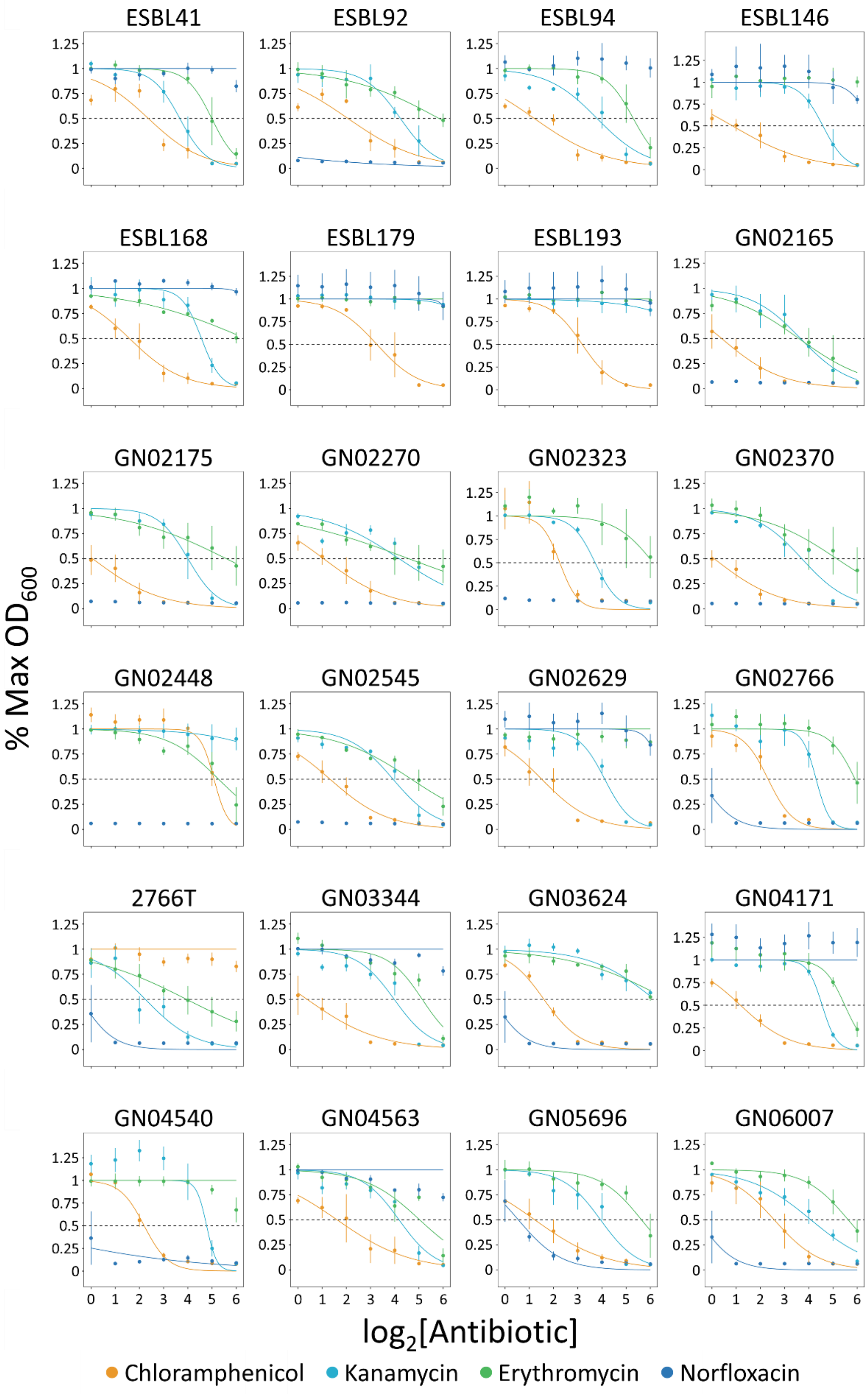

ESBL41
ESBL92
ESBL94
ESBL146
ESBL168
ESBL179
ESBL193
GN02165
GN02175
GN02270
GN02323
GN02370
GN02448
GN02545
GN02629
GN02766
2766T
GN03344
GN03624
GN04171
GN04540
GN04563
GN05696
GN06007
% Max OD_{600}
log_2[Antibiotic]
Chloramphenicol
Kanamycin
Erythromycin
Norfloxacin

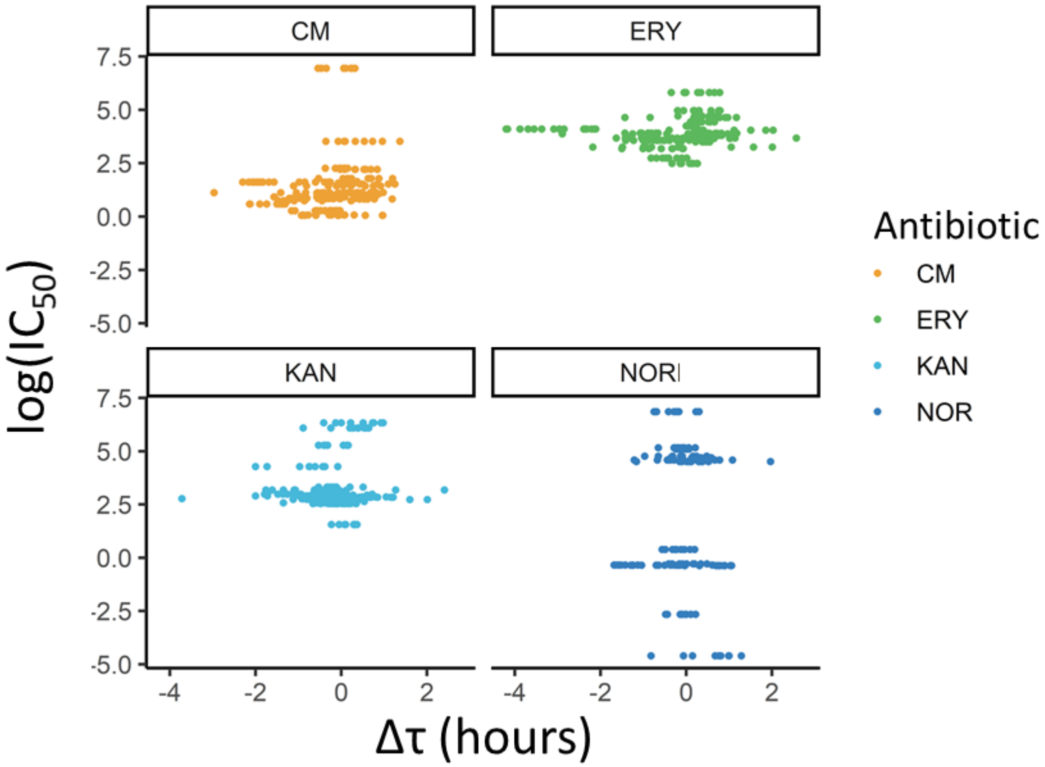

Fig. S3. Antibiotic resistance of plasmid donors. (**a**) Normalized endpoint OD_{600} of pathogen donors under varied antibiotic conditions. Average values from triplicate OD measurements are displayed +/- SE and fit with a two-parameter log-logistic model to estimate IC_{50}. Five antibiotics were tested at concentrations ranging from 0 to 64 µg/mL. The results from carbenicillin are not displayed as every donor displayed the same high-level beta-lactam resistance. (**b**) Strength of antibiotic resistance does not correlate with the effect of antibiotics on τ. IC_{50}s determined for pathogen donors in Figure 3 show no correlation with τ normalized to no antibiotic controls (Δτ) across four antibiotics of varied mechanism of action: chloramphenicol (Cm), erythromycin (Ery), kanamycin (Kan), and norfloxacin (Nor).

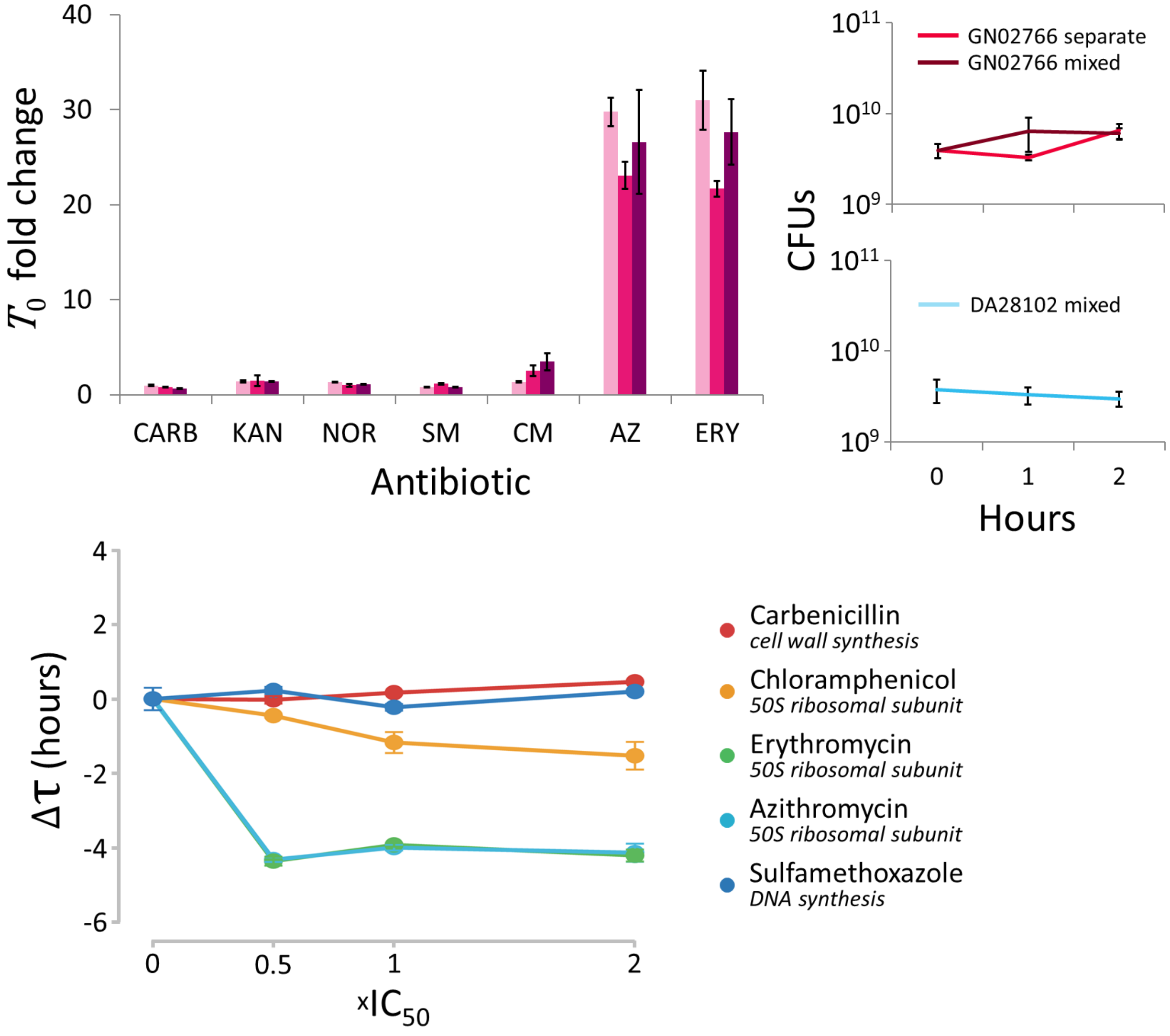

Fig. S4. Antibiotic modulation of conjugation for strain GN02766. (**a**) τ values from antibiotic cultures were normalized by subtracting no antibiotic control τ, then converted into transconjugant cell density using the GN02766 standard curve. Exposure to Carb, Kan, Norf, and Sm resulted in no significant change in transconjugant density, Cm, Az, and Ery increased the number of transconjugants by up to 31-fold. Average normalized T_0 fold change values are displayed with error bars representing +/- SE from triplicate measurements. Bar color darkens with increasing antibiotic concentration. (**b**) Donor-recipient strain interactions for GN02766. GN02766 and DA28102 displayed no significant changes in population density while mixed over two hours under no growth conjugation conditions, relative to separated cultures. This suggests the changes in T_0 are not from donor-recipient interactions that cause growth or death. Error bars represent +/- SE from triplicate measurements. (**c**) Promotion of conjugation in GN02766 is specific to macrolide and chloramphenicol mechanisms of action and not solvent dependent. The effect of two additional bacteriostatic antibiotics on conjugation in GN02766 was assessed via the time to threshold method. Antibiotics were dosed as before (Figure 3), with 1x

concentrations for azithromycin (Az) and sulfamethoxazole (Sm) being 2 μg/mL and 16 μg/mL, respectively. Displayed $\Delta\tau$ are averages of triplicate measurements +/- SE, and normalized by subtracting the no antibiotic control τ. Promotion of conjugation is indicated by $\Delta\tau<0$, while $\Delta\tau>0$ indicates inhibition. Both macrolides, Ery and Az, displayed nearly identical conjugation promotion ($p < 0.0001$, Tukey HSD). Cm, which shares a similar mechanism of action with macrolides, repeated its promotive trend but returned insignificant for this experiment. Sm had no significant effect, indicating conjugation promotion is mechanism-specific, not bacteriostatic-specific. No antibiotic was dosed with EtOH solvent to control for solvent effects.

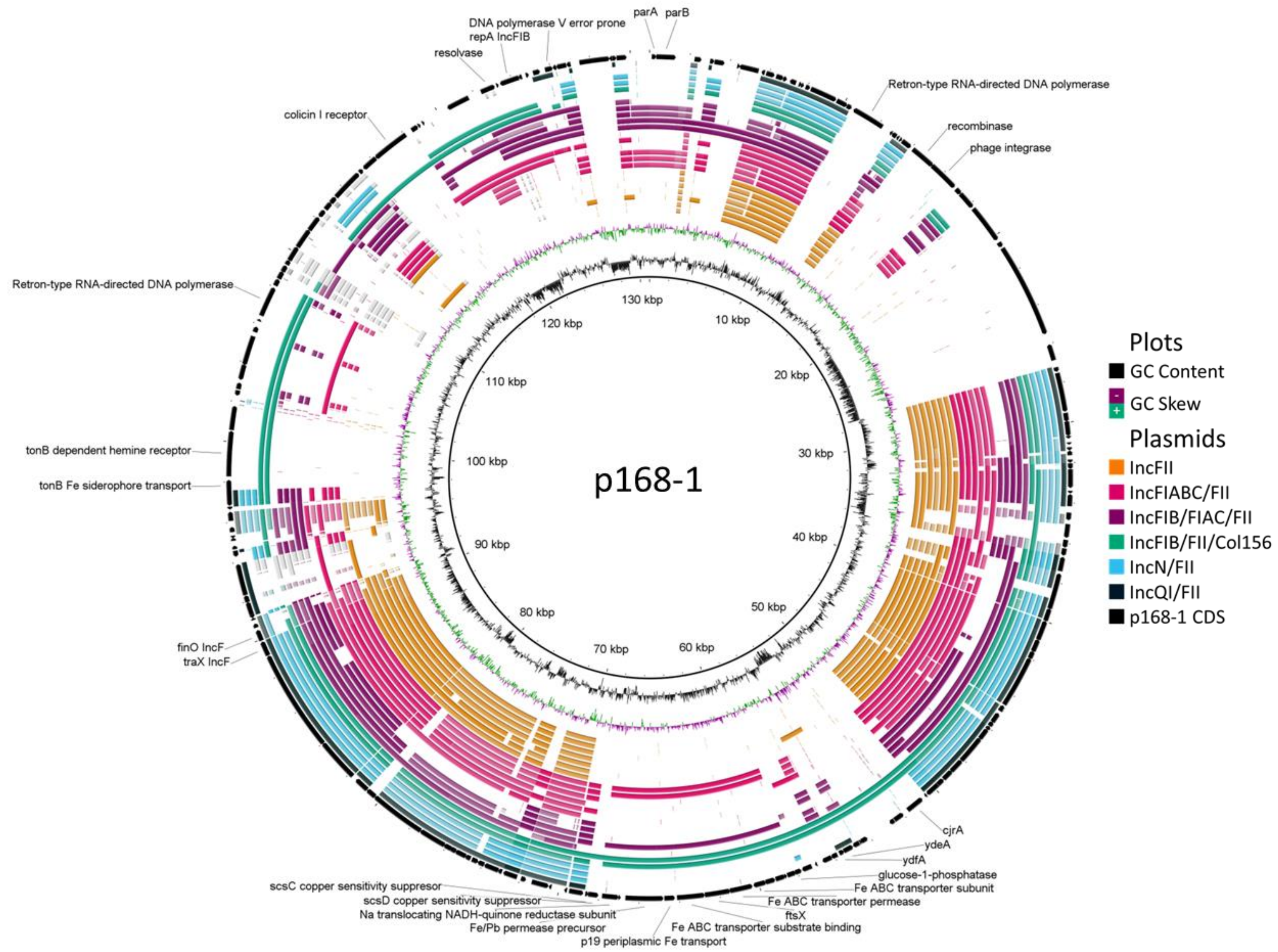
p168-1
Plots
GC Content
GC Skew
Plasmids
IncFII
IncFIABC/FII
IncFIB/FIAC/FII
IncFIB/FII/Col156
IncN/FII
IncQI/FII
p168-1 CDS
130 kbp
10 kbp
20 kbp
30 kbp
40 kbp
50 kbp
60 kbp
70 kbp
80 kbp
90 kbp
100 kbp
110 kbp
120 kbp
parA
parB
DNA polymerase V error prone
repA IncFIB
resolvase
colicin I receptor
Retron-type RNA-directed DNA polymerase
recombinase
phage integrase
Retron-type RNA-directed DNA polymerase
tonB dependent hemine receptor
tonB Fe siderophore transport
finO IncF
traX IncF
cjrA
ydeA
ydfA
glucose-1-phosphatase
Fe ABC transporter subunit
Fe ABC transporter permease
ftsX
Fe ABC transporter substrate binding
p19 periplasmic Fe transport
Fe/Pb permease precursor
Na translocating NADH-quinone reductase subunit
scsD copper sensitivity suppressor
scsC copper sensitivity suppresor

Fig. S5. Identification of plasmid features lost in strain GN02766. Plasmid sequences were aligned to p168-1 via BLASTn and BRIG as the most closely related plasmid to p2766-1 from a no antibiotic modulation strain. Nucleotide identity ≥70% is indicated by a band colored according to Inc group, with darker shading corresponding to higher sequence match. Blank regions indicate <70% nucleotide identity. Inner GC plots, size map, and outer coding sequences (CDS) are for p168-1. The outermost IncFIB/FII/Col156 plasmid corresponds to p2766-1. Among plasmids displaying no antibiotic modulation of conjugation, no major commonalities among the majority were lost in p2766-1. Macrolide and chloramphenicol promotion of conjugation may then be acquired instead.

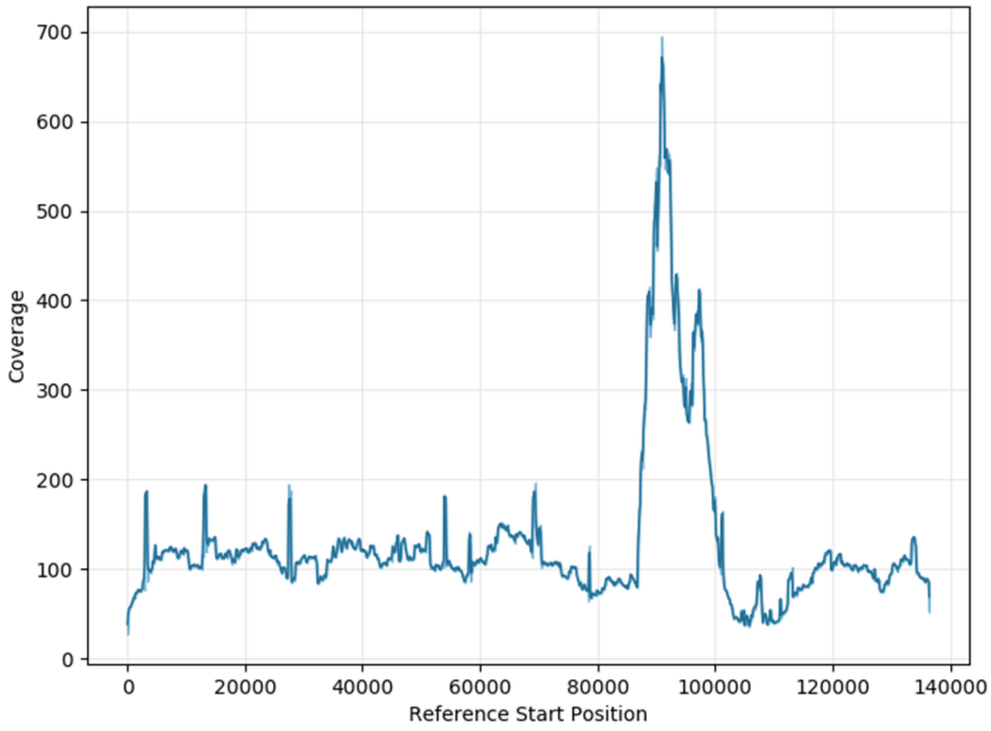

Fig. S6. Sequencing read coverage plot for p2766-1. The 5x tnpA repeats spanning the ~1-25kb region of p2766-1 could be assembly error due to poor read coverage. However, we see no indication of poor coverage across the plasmid, which suggests that the tnpA repeats are genuine. The increase in coverage seen from ~90-100kb is due to a concentration of short reads and not collapsed repeats. This coverage plot was generated through the SMRTLink HGAP4 pipeline with parameters: minMatch = 12; bestn = 10; minPctIdentity = 70.0.

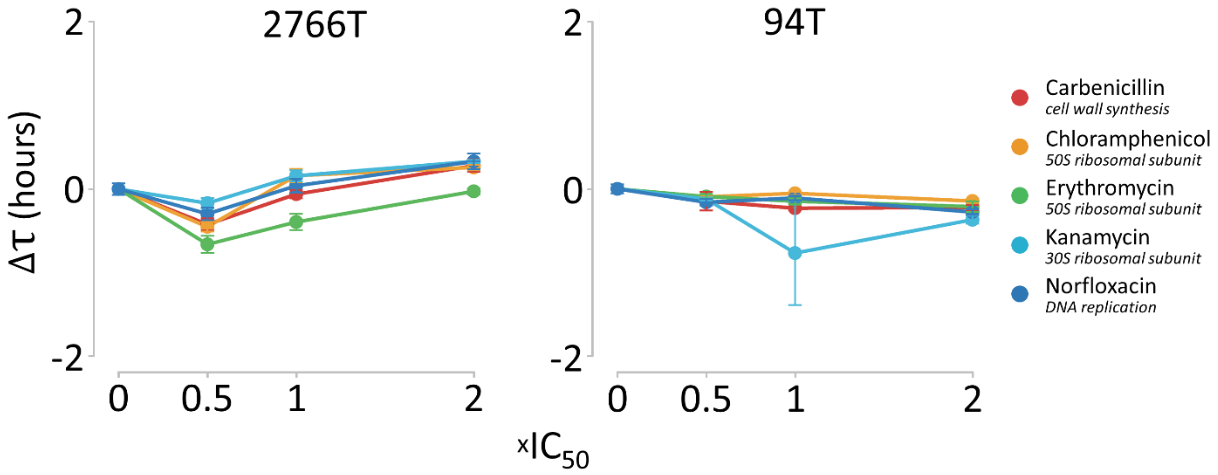

Fig. S7. Macrolide promotion of conjugation is transferrable. (**a**) To measure plasmid-specific effects, 2766T, the DA28102 transconjugant produced by GN02766, was subsequently used as a donor with the fAYC002 recipient. Antibiotics were dosed as before during conjugation (Figure 3). Following conjugation, mixtures were diluted 50x instead of 150x to allow for low conjugation efficiency. Carb100 and Kan50 were used for selective outgrowth for fAYC002 transconjugants. Displayed $\Delta\tau$ are averages of triplicate measurements +/- SE, and normalized by subtracting the no antibiotic control τ. Promotion of conjugation is indicated by $\Delta\tau<0$, while $\Delta\tau>0$ indicates inhibition. Ery once again displayed significant conjugation promotion at both 0.5x and 1x IC_{50} concentrations ($p < 0.0001$ and $p < 0.05$ respectively, Tukey HSD). (**b**) Macrolide promotion is specific to 2766T, not DA28102 transconjugants in general. In line with ESBL94, 94T also displayed no antibiotic modulation of conjugation when used as a donor with fAYC002 recipients. Carb100 and Kan50 were used for selective outgrowth for fAYC002 transconjugants. Displayed $\Delta\tau$ are averages of triplicate measurements +/- SE, and normalized by subtracting the no antibiotic control τ. Promotion of conjugation is indicated by $\Delta\tau<0$, while $\Delta\tau>0$ indicates inhibition.

Table S1. All strains used in this study (*9*, *20*, *49*).

Name	Genotype	Description	Antibiotic Resistance
DA28102	*E. coli*, K-12 MG1655 F- λ- *ilvG-rfb-50 rpb-1 galK::cat*-J23101-mTagBFP2	Plasmid free recipient strain used by Lopatkin et al. (*9*)	Cm
fAYC002	*E. coli*, *K*-12 MG1655 *PRO ΔcsgA ompR234*	Plasmid free recipient strain created by Chen AY et al. (*49*)	Kan, Spec
ESBL#	*E. coli*, Mixed	7 ESBL donors isolated from hospitals associated with the Duke Infection Control Outreach Network in North Carolina and Virginia. See Chen LF et al. (*20*)	Carb, Mixed
GN#	*E. coli*, Mixed	219 isolate library from the Duke Bloodstream Infection Bio-repository over 2002-2014. Beta-lactamase-producing strains used as donors.	Mixed
#T	*E. coli*, DA28102 genotype unless specified	Transconjugants from beta-lactamase donors and either DA28102 or fAYC002 recipients.	Mixed

Table S2. Plasmid composition of pathogenic *E. coli* isolates.

Strain	MOB Relaxase	Plasmid Replicon	Plasmid Size (kb)	Antibiotic Resistance
ESBL41	MOBF	IncFIB/IncFIA/IncFII	121	Phenicol
	MOBF	IncN/IncFII	120	Beta-lactam, Phenicol
EBSL92	MOBP	IncFII/IncI1	82	Beta-lactam
ESBL94	MOBF	IncFIB/IncFIA/IncFII	121	Phenicol
	MOBF	IncN/IncFII	119	Beta-lactam, Phenicol
ESBL146	MOBF	IncFIB/IncFIA/IncFII	100	Aminoglycoside, Beta-lactam, Macrolide, Sulfonamide, Tetracycline
	MOBF	IncFII	69	-
	MOBF	IncN	46	-
	-	IncY	97	-
	-	-	37	-
ESBL168	MOBF	IncFIB/IncFII/Col156	131	Phenicol
	MOBP	IncB/O/K/Z	97	Beta-lactam, Sulfonamide
	MOBF	IncQ1/IncFII	92	Sulfonamide, Tetracycline
	MOBF	IncN2	57	-
	MOBF	IncN2	34	-
	-	IncFIB	109	-
	-	-	93	-
	-	IncI2	60	-
EBSL179	MOBF	IncFIA/IncFII	122	Aminoglycoside, Sulfonamide, Tetracycline
	MOBP	IncI1	91	Aminoglycoside, Beta-lactam
	MOBF	IncFII	72	Beta-lactam
ESBL193	MOBF	IncFIA/IncFII	122	Aminoglycoside, Sulfonamide, Tetracycline
	MOBP	IncI1	91	Aminoglycoside, Beta-lactam

Strain	MOB Relaxase	Plasmid Replicon	Plasmid Size (kb)	Antibiotic Resistance
GN02175	MOBF	IncFIB/IncFII/Col156	145	Phenicol
	MOBP	IncB/O/K/Z	86	Beta-lactam
	MOBQ	IncX1	34	-
	-	IncFII	29	-
	-	-	10	Beta-lactam
GN02448	MOBF	IncFIB/IncFIA/IncFII	124	Beta-lactam, Phenicol, Sulfonamide
	MOBF	IncN*	54	-
	-	p0111	98	-
GN02545	MOBF	IncFII	79	Beta-Lactam
GN02629	MOBF	IncFIA/IncFII	110	Aminoglycoside, Beta-lactam, Macrolide, Sulfonamide, Tetracycline
	-	IncFIB	109	-
GN02766	MOBF	IncFIB/IncFII/Col156	137	Aminoglycoside, Beta-lactam, Sulfonamide
	-	-	29	-
GN03409	MOBF	IncFII	77	Beta-lactam
	MOBF	IncFIB/IncFII	76	-
	-	-	103	-
	-	-	72	-
	-	-	63	-
	-	-	54	-
	-	-	29	-
	-	-	29	-

Strain	MOB Relaxase	Plasmid Replicon	Plasmid Size (kb)	Antibiotic Resistance
GN03624	MOBF	IncFIB/IncFIC(FII)	191	-
	MOBP	IncI1	112	Aminoglycoside, Beta-lactam, Sulfonamide
	-	IncFII	119	Aminoglycoside, Sulfonamide, Tetracycline
	-	-	109	-
	-	-	95	-
	-	-	94	-
	-	-	91	-
	-	-	90	-
	-	-	61	-
	-	-	53	-
	-	-	27	-
	-	-	25	-
	-	-	12	-
GN04540	MOBP	IncFII(pECLA)/IncI1	94	Beta-lactam
	MOBF	IncFII	89	Aminoglycoside, Beta-lactam
	-	-	6	-
GN04563	MOBF	IncFIC(FII)	74	Beta-lactam
	-	IncFIB/IncFIA	44	Tetracycline
GN05696	MOBF	IncN/IncFII	92	Beta-lactam, Sulfonamide
	-	Col	15	-
GN05885†	MOBP	IncB/O/K/Z	120	Beta-lactam, Tetracycline, Sulfonamide
	-	IncFII	79	Beta-lactam, Phenicol
GN06007	MOBF	IncFIB/IncFII	93	Aminoglycoside, Beta-lactam, Sulfonamide, Tetracycline
	MOBF	IncFII(pCoo)	78	-
	MOBF	IncN	32	-

*88% identity †Low sequencing coverage (42x).

Table S3. MICs and IC_{50}s for plasmid donors in this study. Average values in µg/mL from triplicate measurements reported for four of five antibiotics used in Figure 3: chloramphenicol (Cm), kanamycin (Kan), erythromycin (Ery), and norfloxacin (Nor). Carbenicillin was also tested, but is not reported here as all donors had MICs >64 µg/mL. MIC was defined as the first concentration to yield 10% or less of the no antibiotic control's maximum OD_{600}.

Strain	CM		KAN		ERY		NOR	
	MIC	IC_{50}	MIC	IC_{50}	MIC	IC_{50}	MIC	IC_{50}
ESBL41	32	5.16	32	12.7	>64	38.06	>64	-
ESBL92	32	4.19	64	18.82	>64	56.74	1	0.01
ESBL94	32	2.31	64	13.92	>64	39.25	>64	-
ESBL146	16	1.8	64	23.96	>64	39.25	>64	97.46
ESBL168	32	3.01	64	23.64	>64	83.83	>64	116.69
ESBL179	32	9.58	>64	195.95	>64	332.07	>64	90.64
ESBL193	32	9.09	>64	438.29	>64	-	>64	108.85
GN02165	8	1.32	64	12.64	64	12.05	1	-
GN02175	8	1.05	64	16.04	>64	48.17	1	-
GN02270	16	2.09	64	18.19	>64	23.96	1	-
GN02323	32	4.79	32	13.17	>64	69.54	8	-
GN02370	8	1.07	32	13.11	>64	35.63	1	-
GN02448	64	33.84	>64	559.9	>64	38.59	1	-
GN02545	16	2.51	64	15.25	>64	25.81	1	-
GN02629	8	2.86	32	17.35	>64	143.29	>64	90.67
GN02766	16	4.99	32	19.75	>64	60.28	2	0.71
2766T	>64	1031.25	32	4.74	>64	15.49	2	0.75
GN03344	8	1.31	32	15.78	>64	35.22	>64	944.78
GN03624	8	3.04	>64	72.03	>64	103.26	2	0.7
GN04171	8	2.26	64	23.46	>64	44.82	>64	162.63
GN04540	32	4.58	64	27.38	>64	111	2	0.07
GN04563	32	3.08	64	18.13	>64	32.77	>64	173.98
GN05696	32	2.35	32	15.41	>64	49.81	16	1.47
GN06007	32	5.96	64	17.05	>64	49.55	2	0.69

Model development and assumptions

Our model of transconjugant outgrowth consists of three ordinary differential equations that account for growth and conjugation dynamics of the donor (D, 1), recipient (R, 2), and transconjugant (T, 3) populations. There are several parameters, including: d, death rates for each population, μ_T, transconjugant growth rate, N_m, culture carrying capacity, and η, rates for D - R and T - R conjugation.

$$\frac{dD}{dt} = -d_D D \quad (1)$$

$$\frac{dR}{dt} = -d_R R - \eta DR - \eta TR \quad (2)$$

$$\frac{dT}{dt} = -d_T T + \mu_T T\left(1 - \frac{D+R+T}{N_m}\right) + \eta DR + \eta TR \quad (3)$$

In describing the dynamics of these three cell populations, we make several simplifying assumptions. The first being that, experimentally, we use antibiotic concentrations so high that degradation has a negligible effect on d_D, d_R, and d_T. Furthermore, parental cell growth rates are insignificant relative to death rates under strong antibiotic selection. Finally, we assume that η does not change with the strain donating the plasmid (either parental donor or its transconjugant).

Parameter values

Parameters values and initial population sizes were chosen to mimic time to threshold experimental results. N_m was found to be ~10^9 cells/mL, which was set to 1 for simplicity and served as the basis for other constants in the model. Both d_D and d_R were set to 0.5 to ensure significant parental death within a few hours, as found in preliminary tests. Doubling growth is expected during exponential phase, therefore μ_T = 1 with low relative $d_T = 10^{-4}$. Conjugation rate constants (e.g. η = 0.1) were based on experimental donor and recipient cell densities (~10^7 cells/mL) and the average conjugation efficiency (~10^{-12}) found for *E. coli* in preliminary tests. It then follows that: D = 0.01, R = 0.01 - T, and T was varied about 10^{-5} for sensitivity analysis.
